# Validity and reliability of the lederman Prenatal Self-Evaluation Questionnaire (PSEQ) in Brazil

**DOI:** 10.1186/s12884-021-03959-3

**Published:** 2021-07-02

**Authors:** Janiny Lima e Silva, Matheus de Sousa Mata, Saionara Maria Aires Câmara, Íris do Céu Clara Costa, Kleyton Santos de Medeiros, Ricardo Ney Cobucci, Ana Katherine Gonçalves

**Affiliations:** 1 Health Sciences Postgraduate Program, Federal University of Rio Grande Do Norte (UFRN), Major Laurentino de Morais St 1218/1301, Natal, Rio Grande Do Norte (RN) Brazil; 2 Health School, Federal University of Rio Grande Do Norte (UFRN), Natal, Rio Grande Do Norte (RN) Brazil; 3 Faculty of Health Sciences of Trairi, Federal University of Rio Grande Do Norte, Santa Cruz, Rio Grande do Norte (RN) Brazil; 4 Department of Odontology, Federal University of Rio Grande Do Norte (UFRN), Natal, Rio Grande Do Norte (RN) Brazil; 5 Biotechnology Postgraduate Program and Medical School, Potiguar University, Natal, Rio Grande Do Norte (RN) Brazil; 6 Department of Obstetrics and Gynecology, Federal University of Rio Grande Do Norte (UFRN), Natal, Rio Grande Do Norte (RN) Brazil

**Keywords:** Psychosocial adaptation, Pregnancy, Validation, Reliability, Psychometric, Instrument validity

## Abstract

**Background:**

The Lederman Prenatal Self-Evaluation Questionnaire (PSEQ) is used to assess psychosocial adaptation to pregnancy, labor, childbirth, and maternity. The PSEQ is a tool used in various countries and has been translated into Portuguese; however, it needs to be validated in Brazil. This study aimed to analyze the validity and reliability of the PSEQ in Brazilian pregnant women.

**Method:**

This methodological validity study investigated internal consistency and reliability using Cronbach’s alpha and intraclass correlation coefficients. Construct validity was assessed using Pearson’s correlation between domains and confirmatory factor analysis. To assess concurrent validity, Pearson’s correlation between the different domains of the PSEQ and Prenatal Psychosocial Profile-Portuguese Version (PPP-VP) was determined. The level of significance was set at 5%.

**Results:**

This study included 399 pregnant women in the northeastern region of Brazil. The internal consistency and reliability of the total PSEQ score were high (Cronbach's alpha = 0.89; intraclass correlation coefficient = 0.95). Validity analysis showed positive and significant correlations between all PSEQ domains, ranging from 0.14 to 0.56. Confirmatory factor analysis demonstrated the following values of goodness of fit: RMSEA = 0.05, SRMR = 0.08, CFI = 0.61, χ^2^/df = 1.77. The discriminant and concurrent validities of the PSEQ were confirmed.

**Conclusions:**

The Portuguese version of the PSEQ has adequate psychometric properties and is a valid and reliable tool to evaluate psychosocial adaptation to pregnancy in Brazilian pregnant women.

## Background

The experience of pregnancy in a woman's life affects various aspects of her lifestyle, causing changes in her social role and requiring adjustments in her personal behavior. It is a period of maternal psychosocial adjustment, when women may experience ambiguous feelings towards gestation and motherhood [1], and it has been reported that the pregnancy-puerperal period is associated with a high risk of psychological disorders in women [2].

Anxiety, stress, and depression are some of the most common psychological disorders associated with pregnancy [3]. High levels of stress and anxiety have been associated with adverse maternal and fetal outcomes. In the presence of stress and anxiety, it becomes difficult to adopt a maternal role and the risk of postpartum depression and deterioration of the perceived quality of life increases [4–6]. Consequently, unborn children are highly susceptible to preterm birth [7], low birth weight [8, 9], and neurodevelopmental problems [10]. In short, poor adaptation to pregnancy can generate several negative effects related to anxiety, depression, and maternal concerns during prenatal care [11], which negatively affect the health and well-being of the child during its life course [12].

Therefore, the evaluation and follow-up of maternal psychosocial well-being during pregnancy are important steps in planning strategies for promoting the health of mothers and their newborns in an integrated manner.

Some questionnaires have been developed over the years to investigate the elements of maternal psychosocial adaptation in different cultural settings [13–15]. In the Brazilian context, only two questionnaires are available for this purpose: the Prenatal Psychosocial Profile-Portuguese Version (PPP-VP) [16] and the Lederman Prenatal Self-Evaluation Questionnaire (PSEQ) [17]. The PPP-VP considers four constructs of the psychosocial profile in prenatal care: self-esteem, stress, social support received from one’s partner, and social support received from other people [16]. However, the PPP-VP does not consider aspects of labor and delivery, which are important because they have a considerable impact on the mothers’ perceptions. The PSEQ [1] includes seven dimensions of psychosocial adaptation to pregnancy that evaluate feelings related to pregnancy, labor, delivery, and maternity. The PSEQ shows good validity and reliability in many countries around the world [18–20] and is used in clinical research to analyze maternal psychosocial adaptation in various contexts such as antenatal care, high-risk hospitalized and low-risk mothers [21], antenatal education [22], and anxiety [23], among others. Although it has been culturally adapted for the Brazilian population, its validity and reliability in this population are unknown, limiting its use in clinical practice and research. Thus, the objective of this study was to analyze the validity and reliability of the PSEQ for use in Brazil.

## Methods

This methodological validity study was conducted in the northeastern region of Brazil. Data were collected from the Brazilian public health system.

### Ethical considerations

The study was approved by the Research Ethics Committee of the Federal University of Rio Grande do Norte (CEP-UFRN), Brazil, under approval number 1.065.285/2014. All participants were informed about the research objectives and procedures and provided written informed consent, in accordance with Resolution 466/12 of the National Health Council of Brazil.

### Sample size

A sample size that is five times the number of items on the instrument being validated [24] was recommended; this was increased by 10% to take into account possible losses. Thus, a sample of 409 volunteers who agreed to participate was selected for this study.

### Study participants

**Fig. 1:**
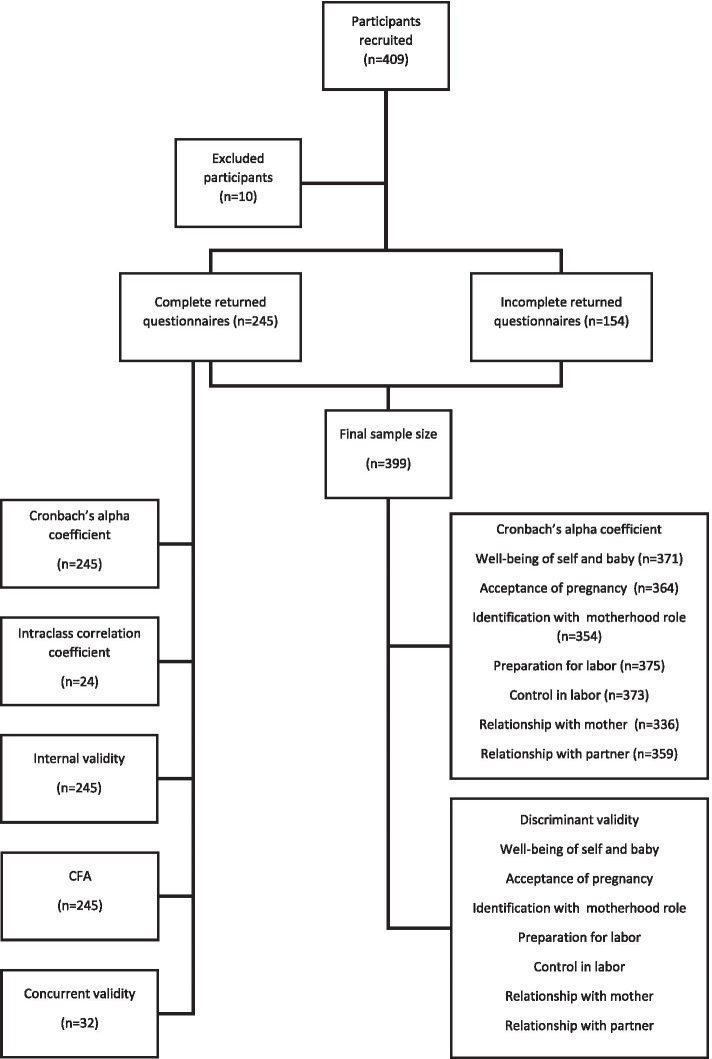
Selection of study participants

The inclusion criteria in this study were as follows: Brazilian citizens who received prenatal care in one of the public health services mentioned above, age at least 14 years, and having a minimum level of education (elementary school). The inclusion criteria were defined according to ethical requirements and relevant heterogeneity samples for methodological analysis [25]. Return questionnaires with no response to one or more items of each dimension of the PSEQ were excluded. Figure 1 shows a flow diagram for the selection of study participants.

The sample size for test–retest evaluation was determined according to the volunteer's availability to attend the Public Health System services during the reevaluation period. A convenience sample was used to determine which women would complete the PPP-VP in addition to the PSEQ, according to the volunteer's availability to remain in the health service as long as necessary to complete both instruments.

### Recruitment and data collection

All pregnant women were invited to participate in the study when they were in the waiting room for prenatal consultation. Initial data collection was performed between October 2014 and July 2015 after obtaining ethical approval for the research, and final data collection was completed between November 2019 and March 2020, as the minimum sample size required for analysis was not reached in the first phase of data collection.

The investigators were trained by the research coordinator and received an instruction manual for data collection. The pregnant women received the evaluation instruments and could respond independently or request the researcher’s help in reading and completing the questionnaires.

First, we collected sociodemographic, clinical, and obstetric data using a questionnaire developed for this study. An additional variable was the Brazilian minimum monthly wage (MW), defined as the lowest remuneration that workers receive as payment for their jobs per month, in addition to variables including age, gestational age, employment status (unemployed or employed), family income (≤ 1 MW or > 1 MW), education level (high school or below, college or above), partner (no or yes), parity (primigravidas or multigravidas), number of appointments with doctors during prenatal care, planned pregnancy (no or yes), and high or low-risk pregnancy based on antenatal care received. Pregnant women were classified as low or high risk according to the criteria used by the Ministry of Health of Brazil [26]. We then administered a version of the PSEQ previously translated and culturally adapted to Brazilian populations by Silva et al. [17]. First, authorization was requested from the author for the translation and validation of the Brazilian Portuguese version of the PSEQ. Two translators fluent in English translated the instrument into Brazilian Portuguese [17]. The translations were then reviewed by an expert committee composed of four people who agreed to the final version of the instrument [17]. Subsequently, the questionnaire was sent to two English-speaking translators for backtranslation [17]. The PSEQ was administered to 36 pregnant women in a pilot study to determine its clarity and coherence [17].

Originally developed in English in 1984, the PSEQ was designed by a North American nurse, Regina Lederman [1]. This assessment tool is meant for pregnant women and includes 79 items in seven dimensions of psychosocial adaptation to pregnancy: Well-being of self and baby (items 12, 16, 17, 30, 41, 51, 57, 63, 68, and 71); acceptance of pregnancy (items 1, 3, 9, 22, 32, 58, 61, 62, 66, 69, 74, 76, 77, and 79); identification with motherhood role (items 2, 6, 19, 29, 33, 34, 42, 45, 46, 50, 54, 67, 73, 75, and 78); preparation for labor (items 7, 13, 24, 25, 26, 38, 47, 48, 56, and 72); control in labor (items 8, 11, 15, 18, 27, 39, 49, 52, 53, and 64); relationship with mother (items 14, 20, 21, 28, 31, 37, 44, 55, 59, and 65); and relationship with partner (items 4, 5, 10, 23, 35, 161 36, 40, 43, 60, and 70). Each item consists of a statement related to the period of pregnancy, childbirth, and maternity to which the respondents need to indicate their degree of agreement using a four-point Likert scale, with the following options: (4) very much so, (3) moderately so, (2) somewhat so, and (1) not at all. However, the scoring for questions with positive statements (questions 1, 2, 3, 4, 6, 7, 8, 10, 11, 12, 14, 15, 18, 19, 20, 21, 22, 23, 24, 25, 26, 28, 31, 32, 33, 35, 36, 37, 38, 40, 47, 48, 49, 50, 53, 55, 56, 59, 60, 61, 70, 71, 72, 73, 74, 75, 78, and 79) was reversed. High scores indicate poor adaptation, whereas low scores indicate increased adaptation. The final score allows the assessment of psychosocial adaptation to pregnancy by means of a specific result, with a total score ranging from 79 to 316.

In 1994, a group of North American nurses developed the Prenatal Psychosocial Profile (PPP) [27]_,_ which was adapted and translated in Brazilian Portuguese and then designated the Prenatal Psychosocial Profile-Portuguese Version (PPP-VP) [16]. This tool has four subscales, each with 11 items: stress, support received from the partner, support received from other people, and self-esteem, totaling 44 items [16]. Scores ranges from 1 to 4 for stress and self-esteem and 1 to 6 for support received from the partner and support received from other people, with high ratings reflecting positive adaptation, and low scores poor adaptation [16].

Participants whose mothers were deceased and those who did not have a partner did not respond to the items related to the relationship with mother and relationship with partner or support received from the partner, respectively.

### Statistical analyses

Descriptive statistics were used to characterize the sample using medians and standard deviations for continuous variables and absolute numbers and relative frequencies for categorical variables.

To determine reliability, internal consistency was evaluated using Cronbach’s alpha coefficient. To calculate the intraclass correlation coefficient (ICC), a test–retest evaluation was conducted with 24 eligible women with a 1-week interval to ensure the stability of the analysis. A Cronbach’s alpha higher than 0.7 was considered reliable. An ICC ≥ 0.40 is considered good and ≥ 0.75, excellent [28].

Construct validity was assessed using 1) confirmatory factor analysis (CFA), 2) discriminant validity determined using Pearson’s correlation coefficient of the seven domains, 3) internal validity determined using Pearson’s correlation coefficient between domains and the general scores and 4) concurrent validity determined by calculating Pearson’s correlation between the individual domains of the PSEQ and PPP-VP with 32 eligible women, as both instruments evaluate the same construct: prenatal psychosocial adaptation. Generally, the goodness of fit of a model is confirmed by the following indices: root mean square error of approximation (RMSEA) < 0.08, standardized root mean square residual (SRMR) < 0.08, comparative fit index (CFI) ≥ 0.90, and normed chi-square (χ^2^/df) < 5.00 [29, 30]. Correlations between the domains of the PSEQ were analyzed to test discriminant validity [1]. The strength of the Pearson’s correlation coefficient increases both from 0 to + 1, and from 0 to -1, therefore, r < 0.40 is considered weak, 0.40 ≤ r < 0.70 moderate, and r ≥ 0.70 strong [31]. A significance level of 5% was considered statistically significant. Participants who did not respond to one or more items in a given domain were excluded from the analysis of that domain. Data analysis was performed using Statistical Package for Social Sciences (SPSS), version 23 for Windows.

## Results

The total sample consisted of 409 pregnant women; 10 participants were excluded because of incomplete data or strikethrough responses to one or more items in every domain. Of the 399 participants, 245 answered the questionnaire in full (61.4%). Therefore, the final sample size was 399 women (average age 26 years, SD ± 7.0, range 14–45 years). The average family income was 1.8 MW (SD ± 1.2), with an average of 3.5 people in the household (SD ± 1.5). The average gestational age of the participants was 26.5 weeks (SD ± 10.2), and the average number of prenatal consultations was five (SD ± 3.2, range of 1–16). Other sociodemographic, clinical and obstetric characteristics of the study participants are presented in Table 1.

**Table 1:** Characteristics of the overall study sample (*n* = 399) and participants who returned complete questionnaires (*n* = 245)

Characteristics^a^	*n* = 399		*n* = 245	
	n	(%)	n	(%)
Age group				
Adolescents (< 20 years)	72	(18.04)	56	(22.9)
Adults (≥ 20 years)	327	(81.95)	189	(77.1)
Education level				
High school or below	329	(82.46)	191	(77.9)
College or above	70	(17.54)	54	(22.1)
Employment status				
Unemployed	178	(45.18)	109	(45.0)
Employed	216	(54.82)	136	(55.0)
Partner				
No	25	(6.30)	0	(0.0)
Yes	374	(93.70)	245	(100.0)
Planned pregnancy				
Yes	181	(46.17)	114	(47.3)
No	211	(53.83)	127	(52.7)
Parity				
Primigravidas	197	(49.50)	125	(51.0)
Multigravidas	201	(50.50)	120	(49.0)
Antenatal care				
Low risk	221	(55.50)	137	(58.3)
High risk	177	(44.50)	98	(41.7)

^a^Missing responses were excluded from analyses

### Reliability

A Cronbach’s alpha (α) of 0.89 was reported. The ICC for the overall scores between the test–retest evaluations was 0.95 (*p* < 0.001), indicating excellent test–retest reliability. The results of the domain analyses are presented in Table 2. Table 2 shows, among other results, that: 1) identification with motherhood role and preparation for labor had a Cronbach's alpha of ≤ 0.70; 2) all domains showed moderate to high correlation with the general score, highlighting the instrument's internal validity and 3) acceptance of pregnancy, identification with motherhood role, relationship with mother and relationship with partner showed excellent ICC.

**Table 2:** Cronbach’s alpha, Correlations, Intraclass Correlation Coefficients and based Factor loadings of the PSEQ

	Well-being of self and baby	Acceptance of pregnancy	Identification with motherhood role	Preparation for labor	Control in labor	Relationship with mother	Relationship with partner
Factor loadings	0.058- 0.770*	0.185*- 0.723*	0.050- 0.533	0.023- 0.731	0.340*-0.627*	0.267*- 0.815*	0.293*- 0.780*
Cronbach’s alpha (n)	0.76 (371)	0.79 (364)	0.56 (354)	0.64 (373)	0.70 (373)	0.84 (336)	0.82 (359)
Correlations with general scores	0.64*	0.60*	0.57*	0.55*	0.73*	0.62*	0.62*
Intraclass Correlation Coefficient (95% CI); p-value	0.73 (0.38–0.88); 0.001	0.95 (0.89–0.98); < 0.001	0.84 (0.64–0.93); < 0.001	0.58 (0.02–0.82); 0.022	0.72 (0.35–0.88); 0.002	0.96 (0.91–0.98); < 0.001	0.75 (0.39–0.89); 0.001
Mean (n; SD)	25.3 (371;6.2)	22.5 (364;5.7)	23.6 (354;4.2)	20.1 (373;4.3)	21.8 (373; 5.1)	15.9 (336;6.1)	16.1 (359;6.0)

*PSEQ* Prenatal Self-Evaluation Questionnaire, *S.D* Standard Deviation, *ICC* Intraclass Correlation Coefficient

^*^p < 0.01

### Validity

**Fig. 2:**
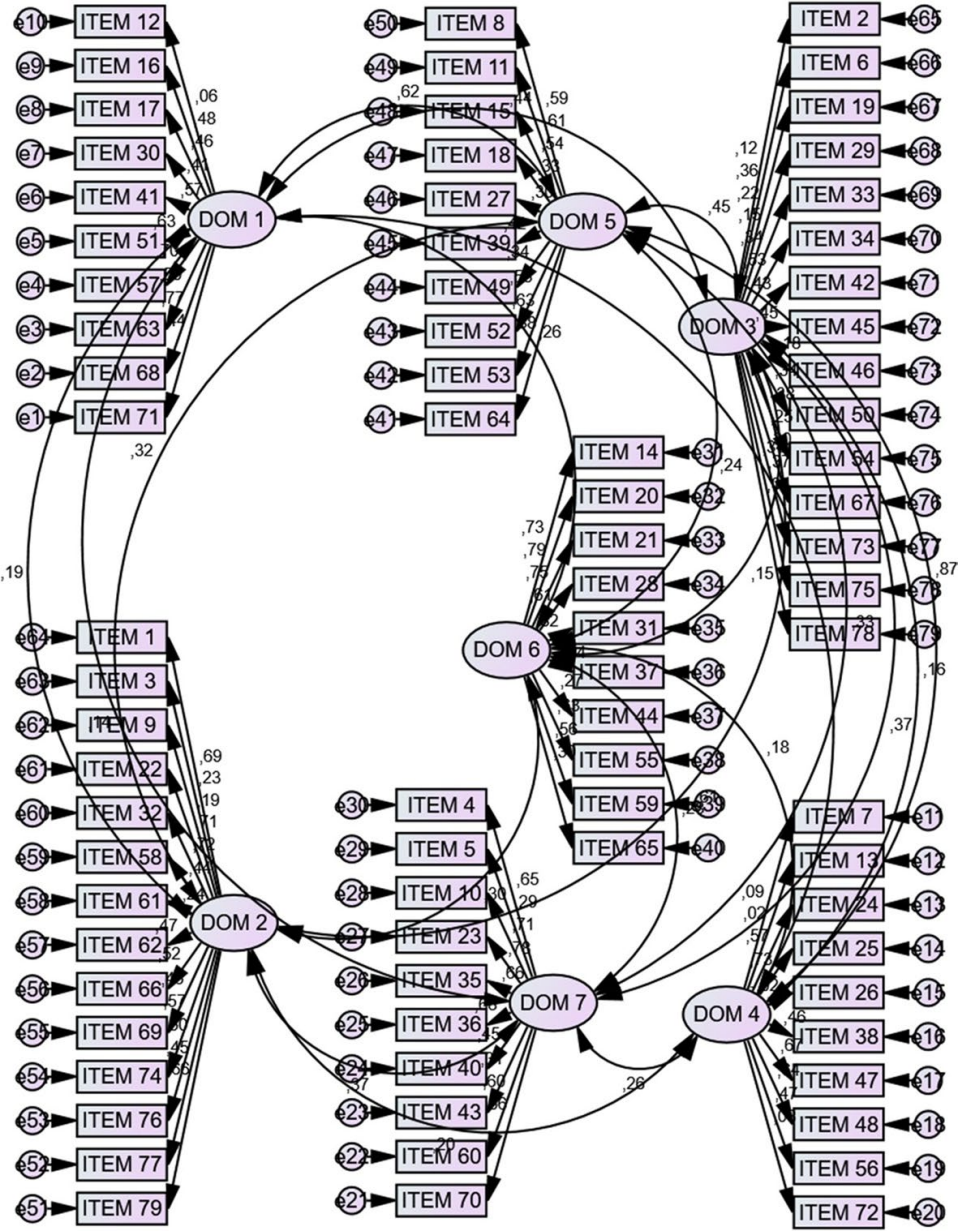
CFA factor loading-Coefficients of item-scale relationship for 79 items of questionnaire (7 ovals represent domains of PSEQ and rectangles represent items of domains, numbers next to the arrows indicate correlation coefficients). DOM 1 = Well-being of self and baby. DOM 2 = Acceptance of pregnancy. DOM 3 = Identification with motherhood role. DOM 4 = Preparation for labor. DOM 5 = Control in labor. DOM6 = Relationship with mother. DOM 7 = Relationship with partner

Confirmatory Factor Analysis (CFA) was carried out to verify the factor structure using the data from women whose questionnaires had no missing answers for any of the domains (*n *= 245), and results are shown in Fig. 2. CFA demonstrated the following values of fitness: RMSEA = 0.05, SRMR = 0.08, CFI = 0.61, χ^2^/df = 1.77.

The internal validity of the correlations between the domains and the general scores of the 245 participants are shown in Table 2. All correlations were significant at *p* < 0.01. Table 3 presents the correlations between PSEQ domains. Discriminant validity analysis showed positive and significant correlations between all PSEQ domains, ranging from 0.14 to 0.56.

**Table 3:** Correlation between PSEQ domains in a sample of Brazilian pregnant women

Domains	Well-being of self and baby	Acceptance of pregnancy	Identification with motherhood role	Preparation for labor	Control in labor	Relationship with mother	Relationship with partner
Well-being of self and baby (n)	1 (372)	0.26* (346)	0.35* (340)	0.20* (355)	0.51* (351)	0.28* (332)	0.19* (350)
Acceptance of pregnancy (n)	-	1 (367)	0.38* (337)	0.14* (353)	0.31* (349)	0.34* (327)	0.42* (345)
Identification with motherhood role (n)		-	1 (357)	0.14* (342)	0.36* (341)	0.22* (324)	0.32* (338)
Preparation for labor (n)			-	1 (375)	0.56* (355)	0.25* (332)	0.29* (353)
Control in labor (n)				-	1 (373)	0.26* (334)	0.29* (350)
Relationship with mother (n)					-	1 (350)	0.34* (324)
Relationship with partner (n)						-	1 (370)

^*PSEQ* Prenatal Self-Evaluation Questionnaire^

^*^p < 0.01

The findings of the present study demonstrated moderate to strong correlations between several domains of PSEQ and PPP-VP, as shown in Table 4. Stress showed a significant positive correlation with acceptance of pregnancy and relationship with partner. Significant negative correlations were observed between the following: acceptance of pregnancy and partner support, other support, and self-esteem; preparation for labor and partner support; control in labor and self-esteem; relationship with partner and partner support, other support, and self-esteem.

**Table 4:** Pearson correlation (p-value) between the domains of the instruments PSEQ and PPP-VP (*n* = 32)

PPP-VP PSEQ	Stress	Support received from the partner	Support received from other people	Self-esteem
Well-being of self and baby	0.23 (0.24)	-0.18 (0.34)	-0.02 (0.91)	-0.08 (0.66)
Acceptance of pregnancy	0.72 (0.00*)	-0.53 (0.00*)	-0.60 (0.00*)	-0.79 (0.00*)
Identification with motherhood role	0.36 (0.06)	-0.05 (0.78)	-0.08 (0.69)	-0.37 (0.06)
Preparation for labor	0.28 (0.13)	-0.42 (0.01*)	-0.32 (0.08)	-0.34 (0.06)
Control in labor	0.33 (0.06)	-0.20 (0.27)	-0.20 (0.26)	-0.57 (0.00*)
Relationship with mother	0.16 (0.41)	-0.34 (0.07)	-0.25 (0.18)	-0.13 (0.48)
Relationship with partner	0.56 (0.00*)	-0.70 (0.00*)	-0.60 (0.00*)	-0.53 (0.00*)

*PSEQ* prenatal self-evaluation questionnaire, *PPP-VP* prenatal psychosocial profile-portuguese version

^*^p < 0.01

## Discussion

The results show that the PSEQ has high internal consistency (Cronbach’s α = 0.89) and is therefore a valid and reliable instrument for a Brazilian population visiting a prenatal public health care provider. Studies carried out in other countries using the same instrument [32, 33] reported similar results: Cronbach’s α of 0.87 (an Iranian study) and 0.93 (a Chinese study). If Cronbach’s α is higher than 0.90, then some items may be duplicated and need to be deleted to ensure internal consistency [34]. The analysis of Cronbach’s α by domain in this study indicated a variation of 0.56–0.84; in other studies that used the instrument in other languages, the variations were 0.68–0.82 [33] and 0.79–0.92 [1]. For the Brazilian sample in this study, identification with motherhood role and preparation for labor showed a Cronbach’s α of < 0.7, which is the recommended minimum value. Similar results were found in a previous study that pre-tested the PSEQ in 36 pregnant Brazilian women [17] with characteristics similar to our sample. The authors also found a Cronbach’s α of < 0.7, in the same domains as the current study. We believe that this finding is related to the different aspects of each domain. The subscale for the domain identification with motherhood role contained a large number of negative statements, which may have resulted in a low coefficient. The low coefficient of preparation for labor could be attributed to the socio-demographic characteristics of the sample, since 18% of the pregnant women were adolescents with low income and low educational levels, who were users of the public health system. In Brazil, women, especially those with low socioeconomic backgrounds, do not traditionally prepare themselves for labor, and this may have interfered with their understanding of the questionnaire statements. A recent study carried out in Brazil showed that knowledge about the type of birth and the use of evidence-based practices during childbirth was low among women with characteristics similar to those of our study sample [35].

The results revealed excellent test–retest reliability between the paired scores of all domains reported before and after the 1-week interval. A study on the PSEQ conducted in Taiwan analyzed its test–retest reliability and found an ICC of 0.95 [32]. Such results could be attributed to the 1-week interval between the evaluations. Test–retest reliability tends to decrease as the retest time is extended [36]. High-to-moderate correlations were observed in each PSEQ domain between evaluation and reevaluation, indicating the reliability of the instrument. Similar to the current study, the test–retest analysis of the original North American instrument showed correlations ranging from 0.67 to 0.82 between the first and third trimesters and from 0.70 to 0.81 between the second and third trimester [1].

CFA results showed acceptable fitness values. The original author did not use factor analysis to determine the construct standards of the scale. The correlations (range 0.14–0.56) between all domains of the PSEQ were positive and significant, confirming the discriminant validity. The strongest correlations were found between well-being of self and baby and control in labor, and lower correlations were found between preparation for labor and both acceptance of pregnancy and identification with motherhood role.

Thus, it may be inferred that greater the well-being of the self and baby, the better the control in labor; the greater the acceptance of pregnancy, the stronger the identification with motherhood role; and the greater the preparation for labor, the greater the control in labor. In a study of the original instrument conducted in the United States [1], the correlations between the PSEQ domains ranged from 0.06 to 0.54, with the strongest correlations reported between well-being of self and baby and control in labor, similar to this study. Correlations were lower between preparation for labor and both acceptance of pregnancy and identification with motherhood role. A similar result was observed in an American study of pregnant women in the second trimester of pregnancy [1]. In the current study, the average gestational age of the participants was 26.5 weeks; therefore, at this stage, there should be a greater interest in preparation for labor even under conditions of a lower acceptance of pregnancy and a lesser identification with the motherhood role.

Lederman and Weiss [1] found that the correlation between the domains was smaller than the reliability of each domain, indicating that the domains are relatively independent, which suggests that a separate analysis is required. Lin et al. [37] analyzed a short version of the PSEQ and found, similar to our study, low-to-moderate correlation, ranging from 0.18 to 0.41, among the subscales, suggesting the presence of discriminant validity between them.

The correlation between the domains and the general score ranged from 0.55 to 0.73, indicating strong to moderate correlation. This result means that all domains of the instrument are part of the evaluated construct, highlighting the internal validity of the instrument. Lin et al. [37] examined the correlation coefficients between the subscales and found a total score of 0.57–0.71.

Finally, the explanatory variables of psychosocial adaptation in both the PSEQ and PPP-VP showed associations as expected, according to the literature. Acceptance of pregnancy and relationship with partner are factors that stand out for maternal psychosocial adaptation during pregnancy. A previous study suggested that among women who reported negative or ambivalent feelings in early pregnancy, unplanned pregnancy was associated with significantly increased odds of psychological distress, while high-quality marital relationships, in particular, reduced the odds of psychological distress [38].

In short, the use of this instrument will help direct an integrated approach to maternal health. In Brazil, health professionals can assess and follow up maternal psychosocial adaptation during prenatal care and develop actions aimed at meeting maternal needs. In other words, identifying concerns, fears, and the quality of support received during pregnancy is an important step in promoting diverse educational actions, such as preparing for labor and encouraging vaginal delivery.

### Strengths and limitations

The PSEQ is a specific and self-administered tool, a valid and reliable instrument that can be used to assess maternal psychosocial adaptation through relevant domains related to pregnancy, labor, childbirth, and motherhood.

This study had some limitations. The reliability and validity of an evaluation instrument must be analyzed in several ways to guarantee its quality [39]. There is a recommendation in the sampling literature for methodological studies that considers a minimum of 5 subjects per variable [24]. However, there is no single sample requirement for all possible analyses. For factor analysis, there is no single criterion for estimating sample size [40]. In general, the sample size may be established as follows: 50 subjects (very poor), 100 subjects (poor), 200 subjects (normal), 300 subjects (good), 500 subjects (very good), and 1000 subjects or more (excellent) [40]. For Hair Jr. et al. (2009), sample sizes between 150 and 400 are suggested [41].

The rate of non-responses in the questionnaires generated a large amount of missing data, which may have influenced the quality of the validation; this stands out as a limitation of the current study that may be related to the presence of domains, such as the relationship with a mother or partner. This fact made it impossible for some participants to complete the questionnaire. Nonetheless, the results showed the instrument's construct validity through the various analyses presented.

The highlight of this study was the heterogeneity of the sample. This may have affected our results. However, heterogeneous samples have been considered a strength in the validation of the instruments [42, 43], as they may facilitate the assessment of the instruments in different clinical and research contexts, when a representative clinical sample is used.

Some volunteers requested the researcher’s help in reading and completing the questionnaires, which may indicate that these participants found the PSEQ too long to complete. Thus, the administration of the instrument through interviews can be useful in populations from low socioeconomic backgrounds. All items were maintained because they were considered relevant to the analysis of psychosocial adaptation to pregnancy.

This study involved women who received prenatal care in public health services. However, considering the diversity of the Brazilian population and voluntary participation in the research, the results may not fully reflect the spectrum of the Brazilian population with regard to sociodemographic characteristics. Of note, the reliability and validity of an instrument can change according to the characteristics of the studied sample; therefore, even an instrument that is already considered valid and reliable should have its validity and reliability tested for each specific sample [36].

## Conclusions

The PSEQ is an instrument that measures psychosocial adaptation to pregnancy in seven domains. After psychometric testing, it demonstrated construct validity, internal consistency reliability, and test–retest reliability. The satisfactory results confirm the reliability and validity of the PSEQ for use in Brazil. The PSEQ will be useful in future research on psychosocial risks and pregnancy outcomes, as well as in clinical practice for antenatal care.

## Data Availability

The datasets used and/or analyzed during the current study are available from the authors on reasonable request. All methods were carried out in accordance with relevant guidelines and regulations in the manuscript.

## Abbreviations

PSEQ: Prenatal Self-evaluation Questionnaire
RN: Rio Grande do Norte
PPP-VP: Prenatal Psychosocial Profile
CEP-UFRN: Research Ethics Committee of the Federal University of Rio Grande do Norte
SPSS: Statistical Package for Social Sciences
S.D: Standard Deviation
MW: Monthly wage
RMSEA: Root mean square error of approximation
SRMR: Standardized root mean square residual
CFI: Comparative fit index
χ^2^/df: Normed chi-square
